# Supplementary material for: Leveraging pleiotropy identifies common-variant associations with selective IgA deficiency
Source: Clin Immunol. Author manuscript; Available in PMC 2026 Jan 6. (PMC7618579; doi:10.1016/j.clim.2024.110356)
Supplement: Supplementary Material [file EMS211685-supplement-Supplementary_Material.pdf]

Appendix A. Supporting information

Supplementary tables

Phenotype	Cases	Controls	Effective sample size	Ancestry	Citation	URL
IgA	0	7,938	7,938	European	This work	
IgA	0	7,677	7,677	European	Dennis et al.	https://ftp.ebi.ac.uk/pub/databases/gwas/summary_statistics/GCST90012001-GCST90013000/GCST90012678/GCST90012678_buildGRCh37.tsv.gz
IgA	0	41,448	41,448	multi-ancestry	Liu et al.	https://www.columbiamedicine.org/divisions/kiryluk/gwas/IgA/META.IGA.LEVELS.ALL.COMBINED.txt
IgAD	1,635	4,852	2,446	European	Bronson et al.	http://ftp.ebi.ac.uk/pub/databases/gwas/summary_statistics/GCST003001-GCST004000/GCST003814/harmonised/27723758-GCST003814-EFO_1001929.h.tsv.gz
IgAD	126	405,620	252	Finnish European	FinnGen	https://storage.googleapis.com/finngen-public-data-r10/summary_stats/finngen_R10_D3_DEF_IIGA.gz
rheumatoid arthritis	35,871	240,149	62,419	multi-ancestry	Ishigaki et al.	https://ftp.ebi.ac.uk/pub/databases/gwas/summary_statistics/GCST90132001-GCST90133000/GCST90132222/GCST90132222_buildGRCh37.tsv.gz
asthma	48,865	371,608	86,372	European	Pan-UKB	https://pan-ukb-us-east-1.s3.amazonaws.com/sumstats_flat_files/categorical-20002-both_sexes-1111.tsv.bgz
Crohn's	12,194	28,072	17,002	European	de Lange et al.	https://ftp.ebi.ac.uk/pub/databases/gwas/summary_statistics/GCST004001-GCST005000/GCST004132/harmonised/28067908-GCST004132-EFO_0000384-build37.f.tsv.gz

juvenile idiopathic arthritis	3,305	9,196	4,862	European	Lopez-Isac et al.	https://ftp.ebi.ac.uk/pub/databases/gwas/summary_statistics/GCST90010001-GCST90011000/GCST90010715/GCST90010715_buildGRCh37.tsv
multiple sclerosis	47,429	68,374	56,007	European	IMSGC	https://imsgc.net/?page_id=31
primary biliary cholangitis	8,021	16,489	10,792	European	Cordell et al.	http://ftp.ebi.ac.uk/pub/databases/gwas/summary_statistics/GCST90061001-GCST90062000/GCST90061440/harmonised/34033851-GCST90061440-EFO_1001486.h.tsv.gz
primary sclerosing cholangitis	4,796	19,955	7,733	European	Ji et al.	https://ftp.ebi.ac.uk/pub/databases/gwas/summary_statistics/GCST004001-GCST005000/GCST004030/harmonised/27992413-GCST004030-EFO_0004268-Build37.f.tsv.gz
rheumatoid arthritis	14,361	43,923	21,645	European	Okada et al.	http://plaza.umin.ac.jp/~yokada/datasource/files/GWASMetaResults/RA_GWASmeta_European_v2.txt.gz
lupus	5,201	9,066	6,610	European	Bentham et al.	https://ftp.ebi.ac.uk/pub/databases/gwas/summary_statistics/GCST003001-GCST004000/GCST003156/harmonised/26502338-GCST003156-EFO_0002690-build37.f.tsv.gz
type 1 diabetes	18,942	501,368	36,505	European	Chiou et al.	http://ftp.ebi.ac.uk/pub/databases/gwas/summary_statistics/GCST90014001-GCST90015000/GCST90014023/harmonised/34012112-GCST90014023-EFO_0001359-Build38.f.tsv.gz
ulcerative colitis	12,366	33,609	18,080	European	de Lange et al.	https://ftp.ebi.ac.uk/pub/databases/gwas/summary_statistics/GCST004001-GCST005000/GCST004133/harmonised/28067908-GCST004133-EFO_0000729-build37.f.tsv.gz
dermatitis/eczema	20,052	198,740	36,429	Finnish European	FinnGen	https://storage.googleapis.com/finngen-public-data-r5/summary_stats/finngen_R5_L12_DERMATITISECZEMA.gz

hyperparathyroidism	2,928	211,123	5,776	Finnish European	FinnGen	https://storage.googleapis.com/finngen-public-data-r5/summary_stats/finngen_R5_E4_HYPERPARA.gz
hypothyroidism	26,064	192,728	45,918	Finnish European	FinnGen	https://storage.googleapis.com/finngen-public-data-r5/summary_stats/finngen_R5_E4_HYTHY_AI_STRICT_PURCH.gz
Addison's disease	1,223	4,097	1,884	Finnish and Norwegian European	Eriksson et al.	http://ftp.ebi.ac.uk/pub/databases/gwas/summary_statistics/GCST90011001-GCST90012000/GCST90011871/GCST90011871_buildGRCh37.tsv.gz
IgA nephropathy	28,751	10,146	14,999	multi-ancestry	Kiryluk et al.	https://www.columbiamedicine.org/divisions/kiryluk/gwas/IgA_Summary/IgAN_Combined_metaanalysis.txt

Supplementary Table 1. The GWAS data sets included in the SIgAD and IgA meta-analyses, and SIgAD cFDR analysis. Pan-UKB is the Pan-UK Biobank study. IMSGC is the International Multiple Sclerosis Genetics Consortium.

Variant	Chromosome	Position	Effect allele frequency	Gene	Novel	IEI	GWAS p-value	Effect size	Effect direction	Study effects
rs16830188:C>T	1	24,972,350	0.02	*RUNX3*			1.36E-64	-0.29	-	--.
rs7551957:T>C	1	161,500,252	0.47	*FCGR2B*	TRUE	TRUE	2.14E-08	0.03	+	...
rs4081545:T>C	1	173,190,377	0.33	*TNFSF4*			1.46E-15	0.04	+	+..
rs7522462:G>A	1	200,912,467	0.26	*INAVA*	TRUE		4.84E-12	-0.04	-	...
rs12713430:A>G	2	60,939,899	0.68	*SANBR*	TRUE	TRUE	6.44E-10	-0.04	-	...

rs13427957:C>T	2	102,072,571	0.44	*IL1R2*			9.66E-09	-0.03	-	-..
rs6859219:C>A	5	56,142,753	0.21	*ANKRD55*			1.88E-27	-0.06	-	-..
rs3777175:A>G	5	95,941,851	0.2	*ELL2*			9.71E-29	0.06	+	+..
rs2153277:C>T	6	45,558,952	0.26	*RUNX2*			1.76E-18	0.05	+	+..
rs9372120:T>G	6	106,219,660	0.21	*PRDM1*	TRUE		2.41E-08	-0.04	-	...
rs17069163:T>C	6	139,654,806	0.2	*CITED2*			1.04E-09	-0.04	-	-..
rs876038:C>T	7	50,268,931	0.32	*IKZF1*	TRUE	TRUE	2.52E-08	-0.03	-	...
rs11486951:A>T	7	76,408,836	0.42	*DTX2*			1.99E-12	-0.03	-	-..
rs3823536:G>A	7	128,939,612	0.46	*IRF5*	TRUE		2.75E-09	0.03	+	...
rs3181356:C>T	9	114,930,602	0.25	*TNFSF8*			5.69E-23	0.06	+	+..
rs968567:C>T	11	61,828,092	0.15	*FADS2*			1.92E-44	0.09	+	+..
rs479844:A>G	11	65,784,486	0.57	*EFEMP2*			1.45E-20	-0.04	-	-..
rs4938518:T>C	11	111,396,669	0.67	*POU2AF1*		TRUE	8.47E-19	-0.04	-	-..
rs7487637:G>A	12	47,821,042	0.27	*HDAC7*			2.87E-12	-0.04	-	-..
rs7137828:C>T	12	111,494,996	0.52	*SH2B3*			3.17E-20	-0.04	-	-..

rs12886625:C>G	14	102,772,597	0.45	*TRAF3*		TRUE	7.14E-13	-0.04	-	-..
rs113962704:T>A	16	11,623,976	0.22	*LITAF*			8.94E-16	-0.05	-	-..
rs1458201:G>A	16	30,904,808	0.25	*CFAP119*			1.56E-11	0.04	+	+..
rs3803800:A>G	17	7,559,652	0.78	*TNFSF13*	TRUE	TRUE	6.75E-11	-0.05	-	...
rs58647797:G>C	17	16,938,289	0.12	*TNFRSF13B*	TRUE	TRUE	2.46E-09	0.05	+	...
rs9625935:A>T	22	30,185,871	0.25	*UQCR10*			1.70E-27	-0.06	-	-..

Supplementary Table 2. Lead SNPs from genome-wide significant associations in the serum IgA GWAS meta-analysis. The 'Variant' column gives the rsID of each SNP, and the reference and effect alleles separated by '>'. Effect allele frequencies given were obtained from gnomAD's estimate in non-Finnish Europeans. 'Gene' gives the gene(s) with the most evidence linking it/them to the association signal. 'Novel' indicates whether an association with SIgAD has previously been reported for a SNP. 'IEI gene' indicates whether the SNP is located in, near, or is otherwise associated with a gene known to harbour variants causal for IEIs. 'GWAS p-value' gives the meta-analytic p-value. 'Effect direction' indicates whether the effect allele is associated with an IgA-increasing ('+') or decreasing ('-') effect. 'Study effects' indicates whether a significant effect was found in the component GWAS of our meta-analysis: Liu, our own GWAS, and Dennis, respectively. '.' indicates no significant effect.

Variant	Chromosome	Position	Effect size (IgA)	Standard error (IgA)	Effect size (SIgAD)	Standard error (SIgAD)	Effect size (IgAN)	Standard error (IgAN)
rs16830188:C>T	1	24,972,350	-0.29	0.02	-0.18	0.24	-0.06	0.05
rs7551957:T>C	1	161,500,252	0.03	0.01	0.02	0.04	-0.10	0.02

rs4081545:T>C	1	173,190,377	0.04	0.01	-0.04	0.04	0.12	0.02
rs7522462:G>A	1	200,912,467	-0.04	0.01	-0.05	0.05	-0.01	0.03
rs12713430:A>G	2	60,939,899	-0.04	0.01	-0.08	0.04	-0.12	0.02
rs13427957:C>T	2	102,072,571	-0.03	0.01	0.11	0.04	-0.02	0.02
rs6859219:C>A	5	56,142,753	-0.06	0.01	0.05	0.05	-0.17	0.03
rs3777175:A>G	5	95,941,851	0.06	0.01	-0.02	0.05	0.00	0.03
rs2153277:C>T	6	45,558,952	0.05	0.01	-0.01	0.05	0.05	0.04
rs9372120:T>G	6	106,219,660	-0.04	0.01	0.13	0.05	0.04	0.04
rs17069163:T>C	6	139,654,806	-0.04	0.01	0.02	0.05	0.01	0.02
rs876038:C>T	7	50,268,931	-0.03	0.01	-0.02	0.05	-0.02	0.02
rs11486951:A>T	7	76,408,836	-0.03	0.00	0.01	0.04	-0.04	0.03
rs3823536:G>A	7	128,939,612	0.03	0.01	-0.02	0.04	0.00	0.02
rs3181356:C>T	9	114,930,602	0.06	0.01	0.00	0.05	0.16	0.04
rs968567:C>T	11	61,828,092	0.09	0.01	-0.10	0.06	0.05	0.03
rs479844:A>G	11	65,784,486	-0.04	0.00	0.02	0.04	-0.09	0.02
rs4938518:T>C	11	111,396,669	-0.04	0.01	0.07	0.04	-0.02	0.02

rs7487637:G>A	12	47,821,042	-0.04	0.01	-0.06	0.05	-0.10	0.04
rs7137828:C>T	12	111,494,996	-0.04	0.00	-0.16	0.04	-0.10	0.03
rs12886625:C>G	14	102,772,597	-0.04	0.00	-0.04	0.04	-0.04	0.02
rs113962704:T>A	16	11,623,976	-0.05	0.01	0.04	0.05	-0.06	0.04
rs1458201:G>A	16	30,904,808	0.04	0.01	0.07	0.05	0.02	0.03
rs3803800:A>G	17	7,559,652	-0.05	0.01	0.01	0.05	-0.14	0.02
rs58647797:G>C	17	16,938,289	0.05	0.01	-0.15	0.07	0.13	0.03
rs9625935:A>T	22	30,185,871	-0.06	0.01	0.08	0.05	-0.16	0.03

Supplementary Table 3. The 'Variant' column gives the rsID of each SNP, and the reference and effect alleles separated by '>'. ‘Effect size’ gives the GWAS effect estimate for the SNP on the parenthetically named phenotype and ‘Standard error’ gives the corresponding standard error of the effect estimate. ‘IgA’ is serum IgA and ‘IgAN’ is IgA nephropathy.

Gene	Analysis	Inborn error of immunity	IgA deficiency
TNFAIP3	SIgAD meta-analysis	Behcet-like autoinflammatory syndrome-1 (A20 deficiency)	no
FAS	SIgAD meta-analysis	autoimmune lymphoproliferative syndrome	no
CD247	SIgAD cFDR	CD3 zeta deficiency (immunodeficiency 25)	no
PTPN2	SIgAD cFDR	CVID	yes
IKZF3	SIgAD cFDR	AIOLOS deficiency	yes
IL2RA	SIgAD cFDR	CD25 deficiency	sometimes
IRF4	SIgAD cFDR	IRF4 haploinsufficiency	no
FCGR3A	IgA meta-analysis	CD16 deficiency (immunodeficiency 20)	no
POU2AF1	IgA meta-analysis	BOB1 deficiency	yes
TRAF3	IgA meta-analysis	TRAF3 deficiency	no
TNFSF12	IgA meta-analysis	TWEAK deficiency (CVID)	yes
TNFRSF13B	IgA meta-analysis	TACI deficiency (CVID)	sometimes
REL	IgA meta-analysis	c-Rel deficiency (immunodeficiency 92)	yes
IKZF1	IgA meta-analysis	IKAROS deficiency (CID/CVID)	sometimes

Supplementary Table 4. ‘Gene’ gives the name of the IEI-associated gene. ‘Analysis’ gives the phenotype with which the gene was associated and the study modality (GWAS meta-analysis or cFDR) used to identify the association in this work. ‘Inborn error of immunity’ gives the IEI with which the gene is associated. ‘IgA deficiency’ indicates whether IgA deficiency is known to be a feature of the IEI.

Variant	Chromosome	Position	Effect allele frequency	Gene	Novel	Effect size	GWAS p-value	Effect direction
rs16830188:C>T	1	24,972,350	0.02	*RUNX3*		-0.28	4.61E-23	-
rs13300483:C>T	9	114,881,082	0.25	*TNFSF8*		0.05	1.82E-08	+
rs117775520:G>A	14	105,707,884	0.04	*IGHA1*		-0.13	8.19E-11	-
rs184702468:C>A	16	26,772,039	0.01	*C16orf82*	TRUE	-0.24	4.41E-08	-
rs5763821:A>C	22	30,153,082	0.38	*HORMAD2*		-0.05	2.95E-09	-

Supplementary Table 5. Lead SNPs from genome-wide significant associations in our GWAS of serum IgA. The 'Variant' column gives the rsID of each SNP, and the reference and effect alleles separated by ‘>’. Effect allele frequencies given were obtained from gnomAD’s estimate in non-Finnish Europeans. 'Gene' gives the gene(s) with the most evidence linking it/them to the association signal. 'Novel' indicates whether an association with serum IgA had previously been reported for a SNP. 'Effect direction' indicates whether the effect allele is associated with an IgA-increasing ('+') or decreasing ('-') effect.

Variant	Chromosome	Position	Gene	Novel	IMD associations	IMD associations in LD
rs1990760:C>T	2	162,267,541	*IFIH1*		hypothyroidism, T1D, UC, psoriasis	vitiligo, T1D, hypothyroidism, SLE, UC, psoriasis
rs9831894:A>C	3	122,081,640	*CD86*	TRUE		
rs2179781:C>A	6	135,398,362	*AHI1*			asthma
rs112920346:C>T	6	137,833,918	*TNFAIP3*	TRUE		T1D
rs72722767:G>A	8	128,192,857	*MYC*			
rs1244181:A>G	10	8,049,414	*GATA3*	TRUE		asthma
rs2031613:C>T	10	89,007,167	*FAS*	TRUE		
rs61882719:A>G	11	46,517,392	*AMBRA1*			
rs34443974:C>T	16	11,085,448	*CLEC16A*		T1D, asthma, MS, eczema	asthma, eczema, T1D, MS, PBC, allergic rhinitis, SLE, nasal polyposis, eosinophilic esophagitis

Supplementary Table 6. The association of the lead SNPs from the SIgAD meta-analysis with other immune-mediated diseases

(IMDs) as identified by GWAS. The 'Variant' column gives the rsID of each SNP, and the reference and effect alleles separated by '>'. 'Gene' gives the gene(s) with the most evidence linking it/them to the association signal. 'Novel' indicates whether an association with SIgAD has previously been reported for a SNP. 'IMD associations in LD' lists IMDs which have significantly associated variants in linkage disequilibrium with the SIgAD-associated variant given in 'Variant'.

rsID	Catalog summary risk allele	Bronson effect allele	Lim effect allele	T2 minor allele	Bronson other allele	Lim other allele	ST2 A allele	ST2 B allele	Catalog summary OR	Bronson OR	Lim OR	T2 OR	ST2 OR	OR A	OR B
rs1990760	T	C	T	G	T	C	C	T	1.4	1.4	1.373376079	0.7	0.7	0.7	1.4
rs34069391	G	GT		1-bp insertion	G		T	G	1.4	1.4		0.71	0.68	0.68	1.5
rs4565870	C	T	C	C	C	T	T	C	1.4	1.4	1.303716458	1.4	1.3	0.76	1.3
rs7773987	T	C	T	C	T	C	C	T	0.7	0.77	0.7721024736	1.3	1.3	1.3	0.76

Supplementary Table 7. ‘Catalog summary’ denotes the summary table of lead SNPs provided on the EBI GWAS Catalog. ‘Bronson’ indicates the GWAS summary statistics downloaded from the EBI GWAS Catalog. ‘Lim’ indicates the GWAS summary statistics we obtained by analysing the genotype data published by Lim et al. ‘T2’ and ‘ST2’ denote Table 2 and Supplementary Table 5 of Bronson et al. ‘OR’ denotes odds ratio. Odds ratios ‘OR A’ and ‘OR B’ were computed from allele counts in Supplementary Table 5 of Bronson et al. taking the ‘A’ and ‘B’ labels as the effect allele, respectively.

Supplementary figures

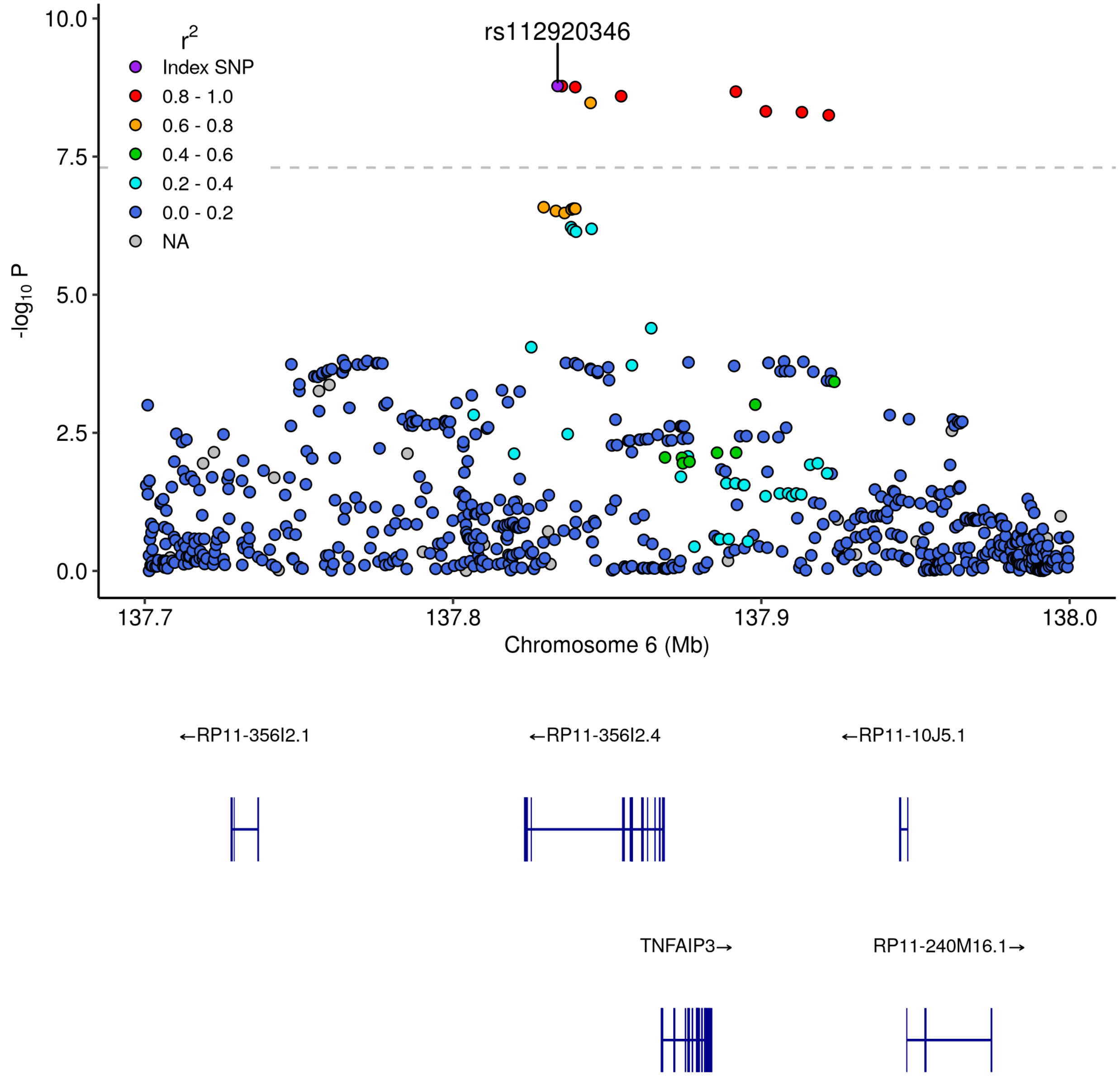

Supplementary Figure 1. The *TNFAIP3* association signal. Each point corresponds to a SNP's p-value from a test of association in the GWAS meta-analysis. The points are coloured according to their squared correlation (r^2) with the lead SNP rs112920346. The *WAKMAR2* gene is RP11-356I2.4.

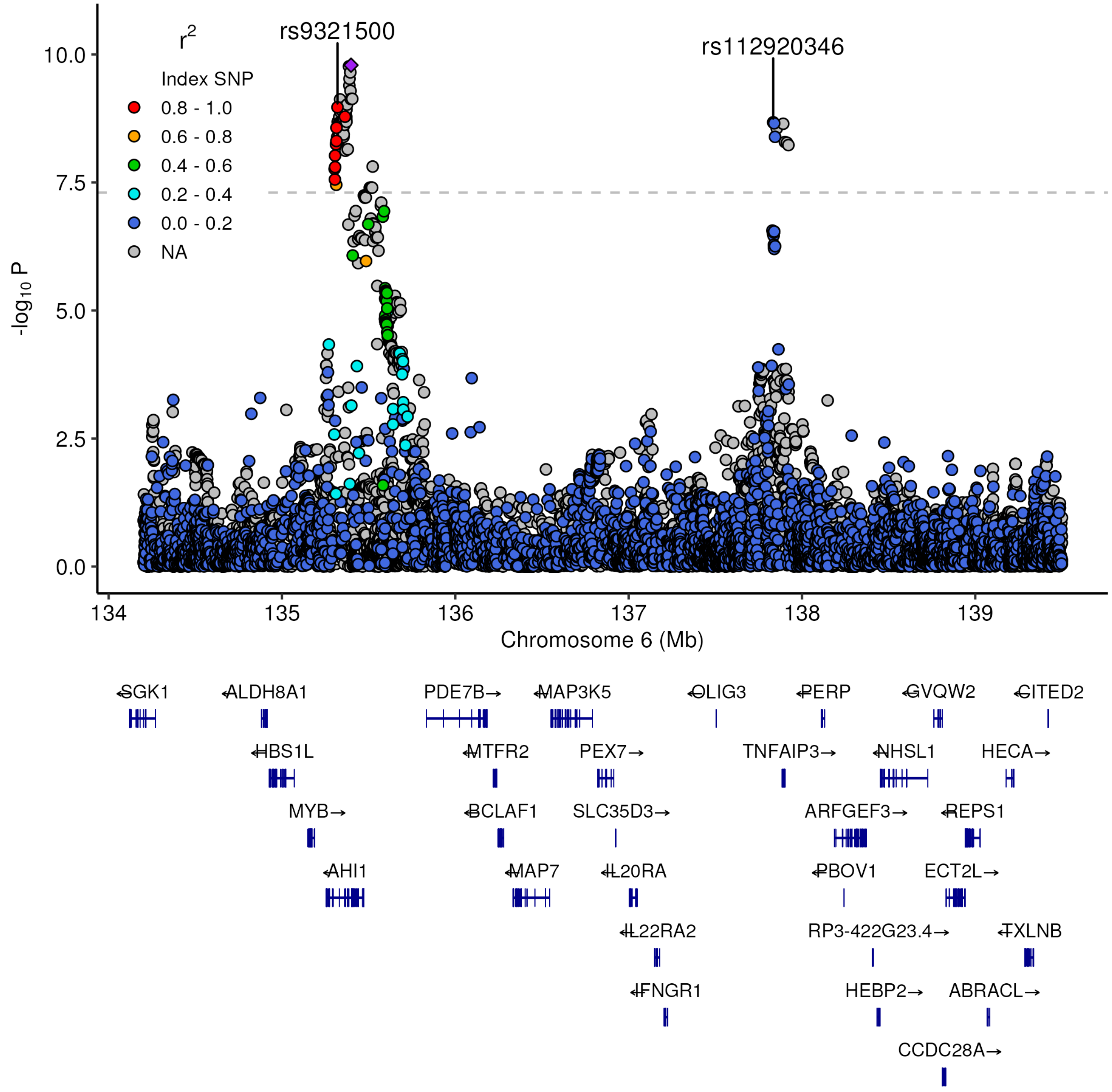

Supplementary Figure 2. The *TNFAIP3* signal was distinct from that which was previously reported at *AHI1*. Each point corresponds to a SNP's p-value from a test of association in the GWAS meta-analysis. The points are coloured according to their squared correlation (r^2) with rs9321500, the SNP in the *AHI1* signal for which LD information was available and which had the smallest p-value. SNPs for which LD information was not available are coloured grey. Only protein-coding genes are depicted.

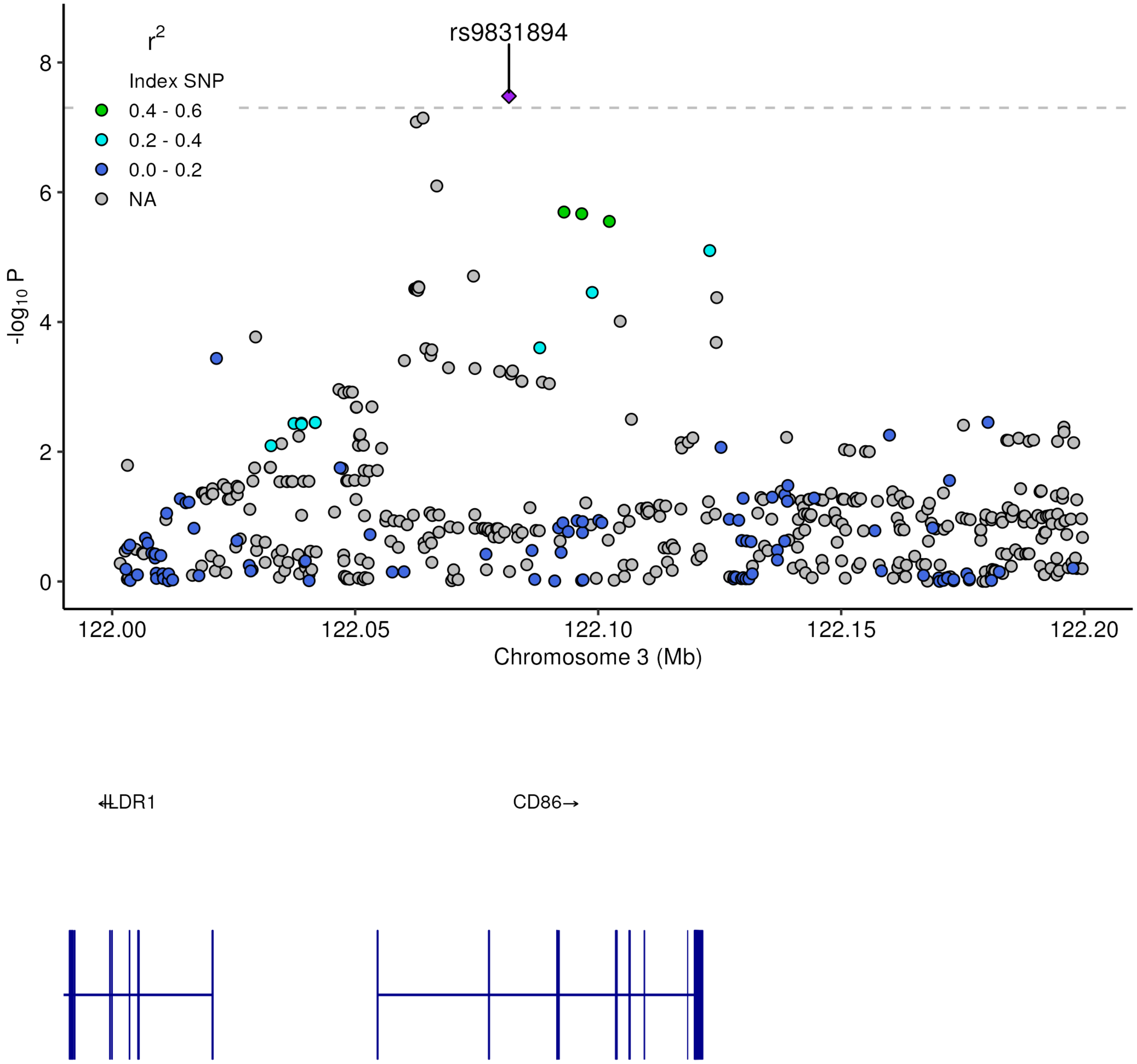

Supplementary Figure 3. The *CD86* GWAS association signal. Each point corresponds to a SNP's p-value from a test of association in the GWAS meta-analysis. The points are coloured according to their squared correlation (r^2) with the lead SNP rs983189.

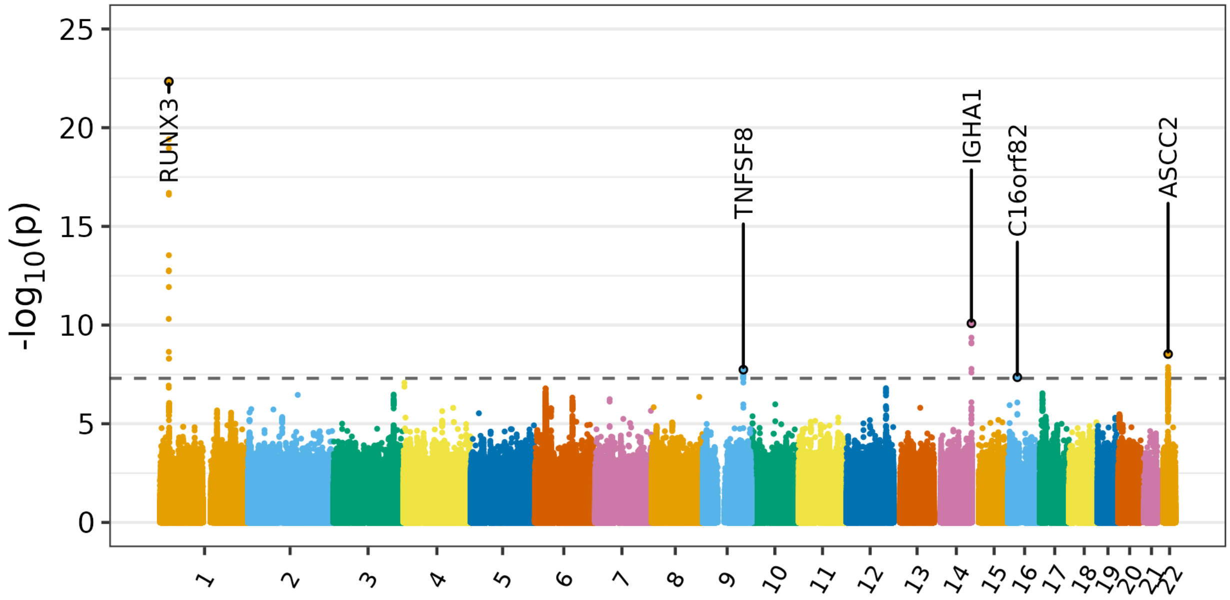

Supplementary Figure 4. Manhattan plot depicting the results of our GWAS of serum IgA. Association signals are labelled with the genes to which they were mapped based on proximity and functional data.

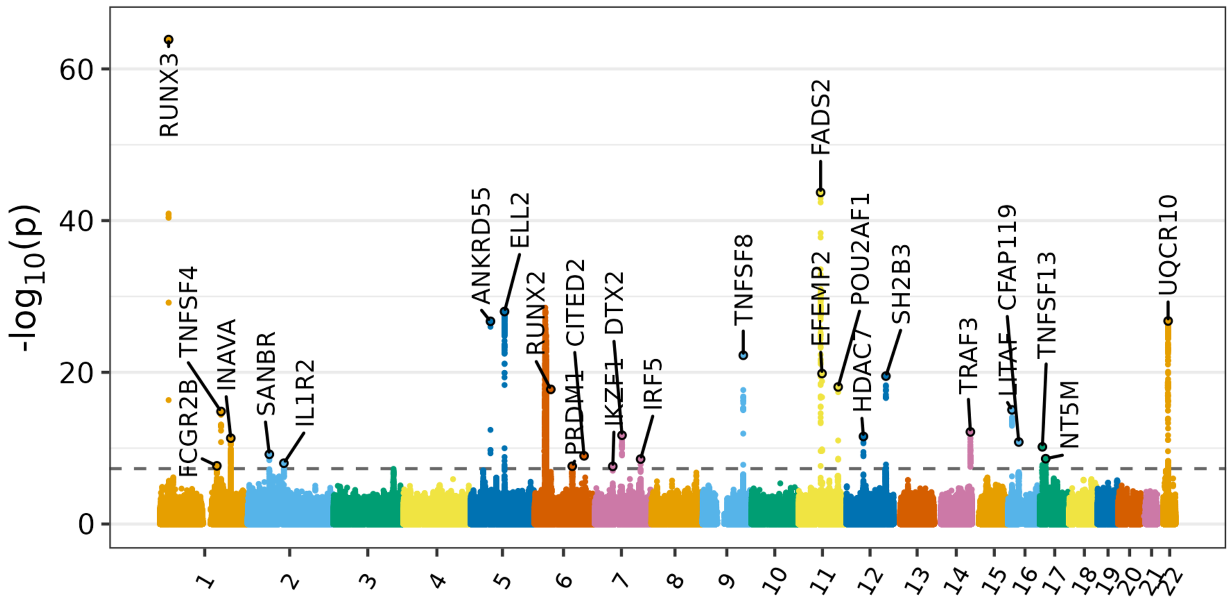

Supplementary Figure 5. Manhattan plot depicting the results of the GWAS meta-analysis of serum IgA. Association signals are labelled with the genes to which they were mapped based on proximity and functional data.

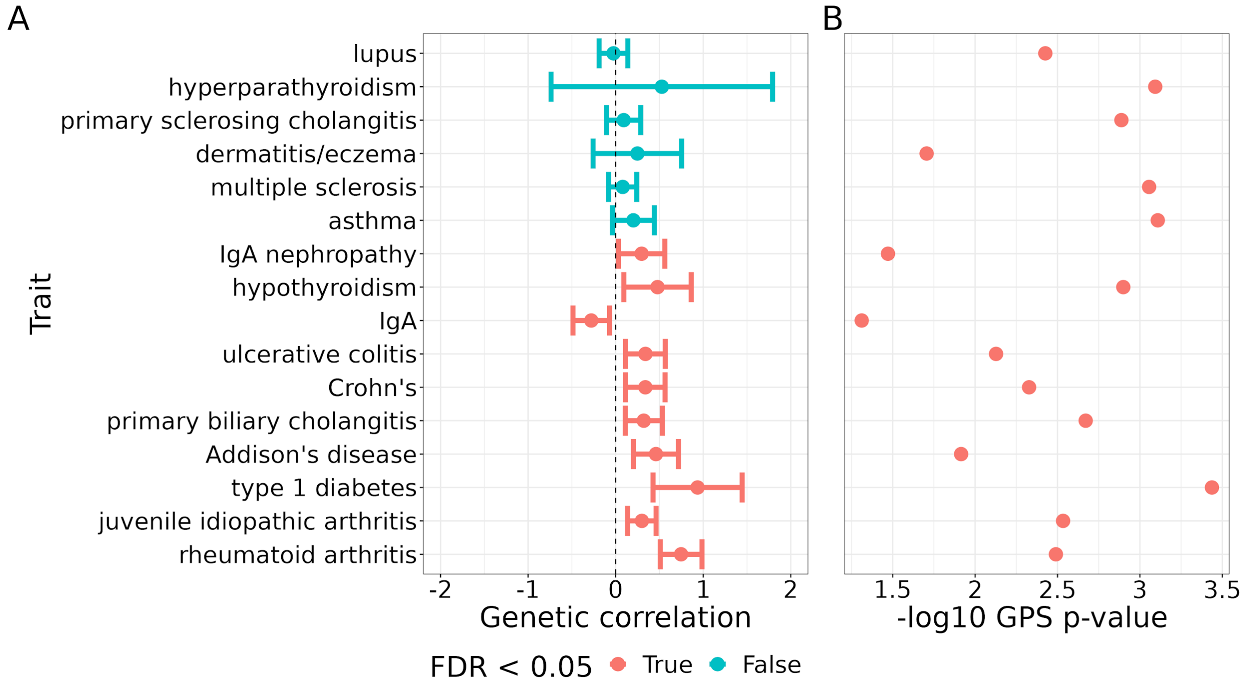

Supplementary Figure 6. Estimates of SIgAD's genetic correlation with a panel of immune traits and p-values from application of the GPS test to SIgAD and members of the same panel. ‘A’ depicts the genetic correlation estimates with 95% confidence intervals. Traits are ordered by the (uncorrected) p-value of a test of non-zero genetic correlation with SIgAD. The colour of the point indicates whether the null was rejected for a test of non-zero genetic correlation with SIgAD (A) or the GPS test (B) whilst controlling FDR at 0.05.

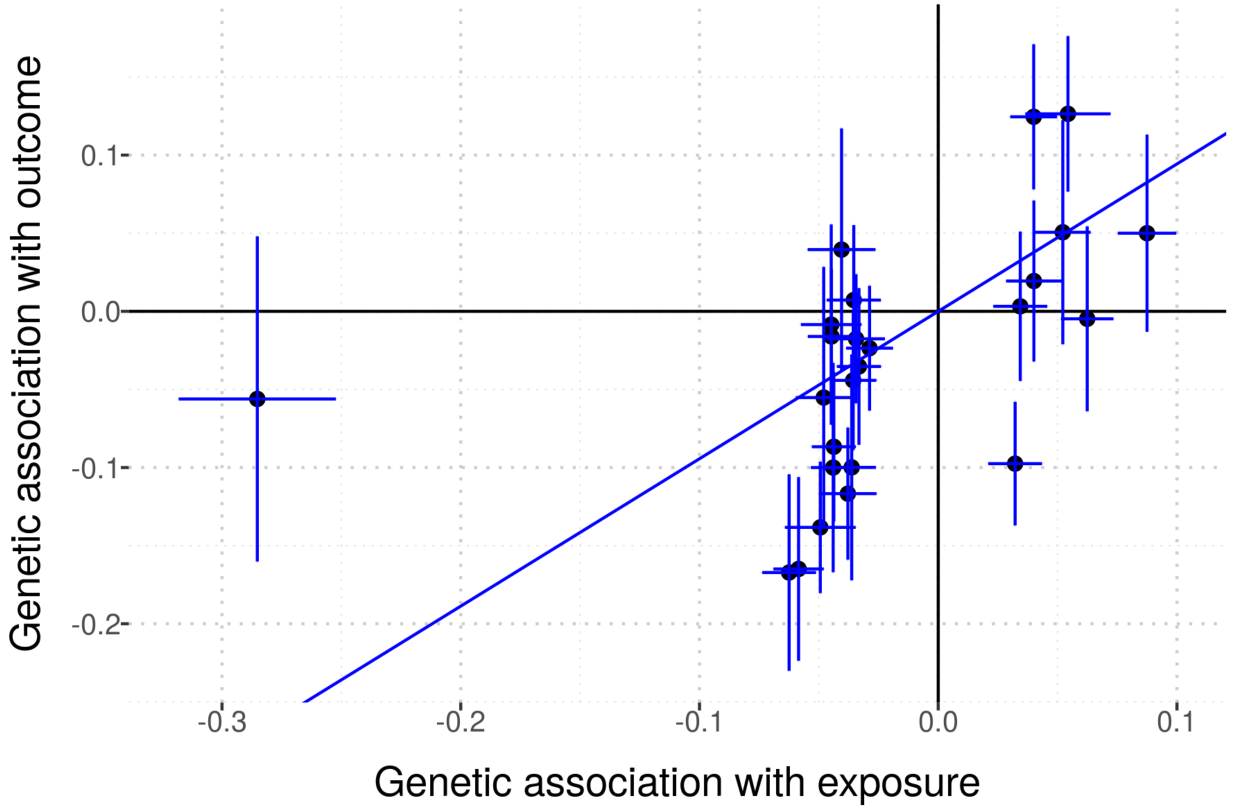

Supplementary Figure 7. Mendelian randomisation of serum IgA (the exposure) and IgA nephropathy (the outcome). Each point corresponds to a SNP which was significantly associated with serum IgA in our meta-analysis. The x-coordinate is given by the effect estimate of the SNP for serum IgA and the y-coordinate by its effect estimate for IgA nephropathy. The blue lines give 95% confidence intervals for the effect estimates for each phenotype. The gradient of the line passing through the origin gives the estimate of the causal effect of serum IgA on the risk of IgA nephropathy, 0.94 ($p < 0.00034$).

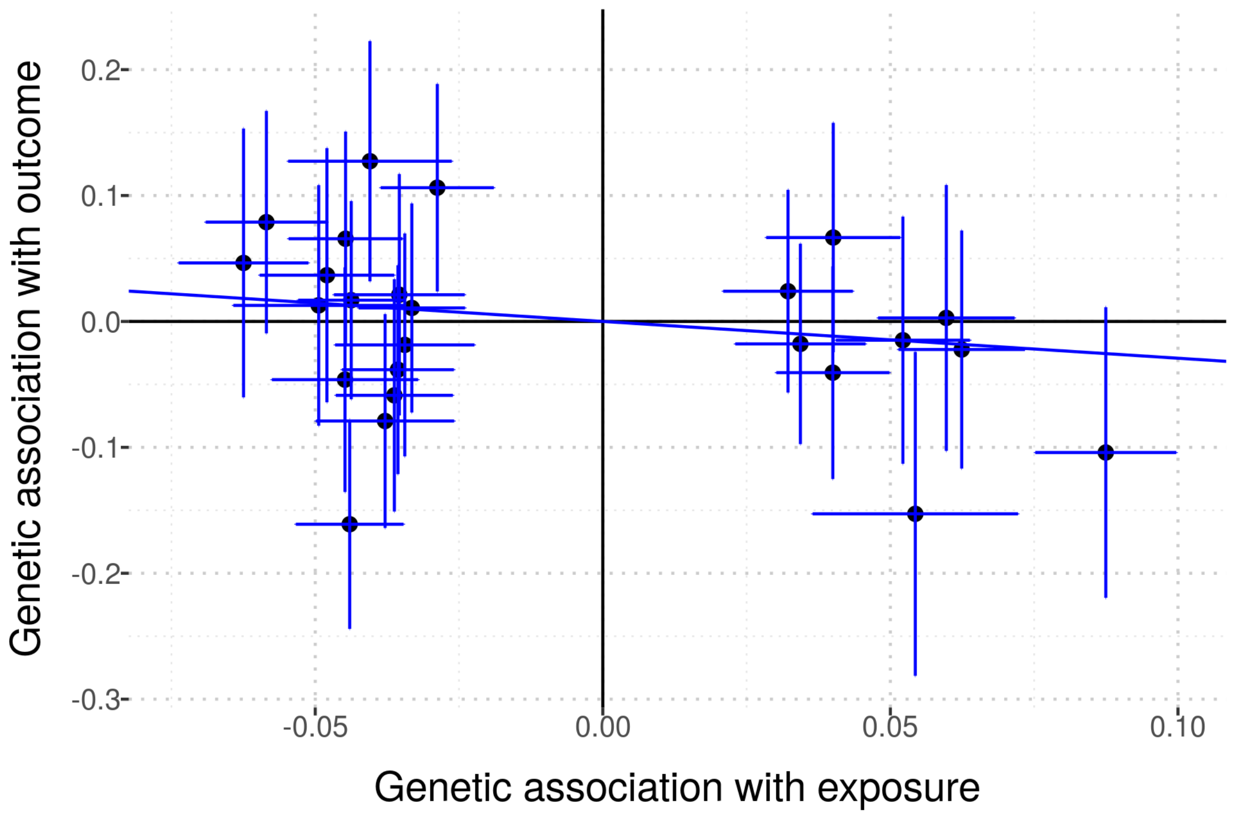

Supplementary Figure 8. Mendelian randomisation of serum IgA (the exposure) and SIgAD (the outcome). Each point corresponds to a SNP which was significantly associated with serum IgA in our meta-analysis. The x-coordinate is given by the effect estimate of the SNP for serum IgA and the y-coordinate by its effect estimate for SIgAD. The blue lines give 95% confidence intervals for the effect estimates for each phenotype. The gradient of the line passing through the origin gives the estimate of the causal effect of serum IgA on the risk of SIgAD, -0.29, which was not significant (p = 0.33).

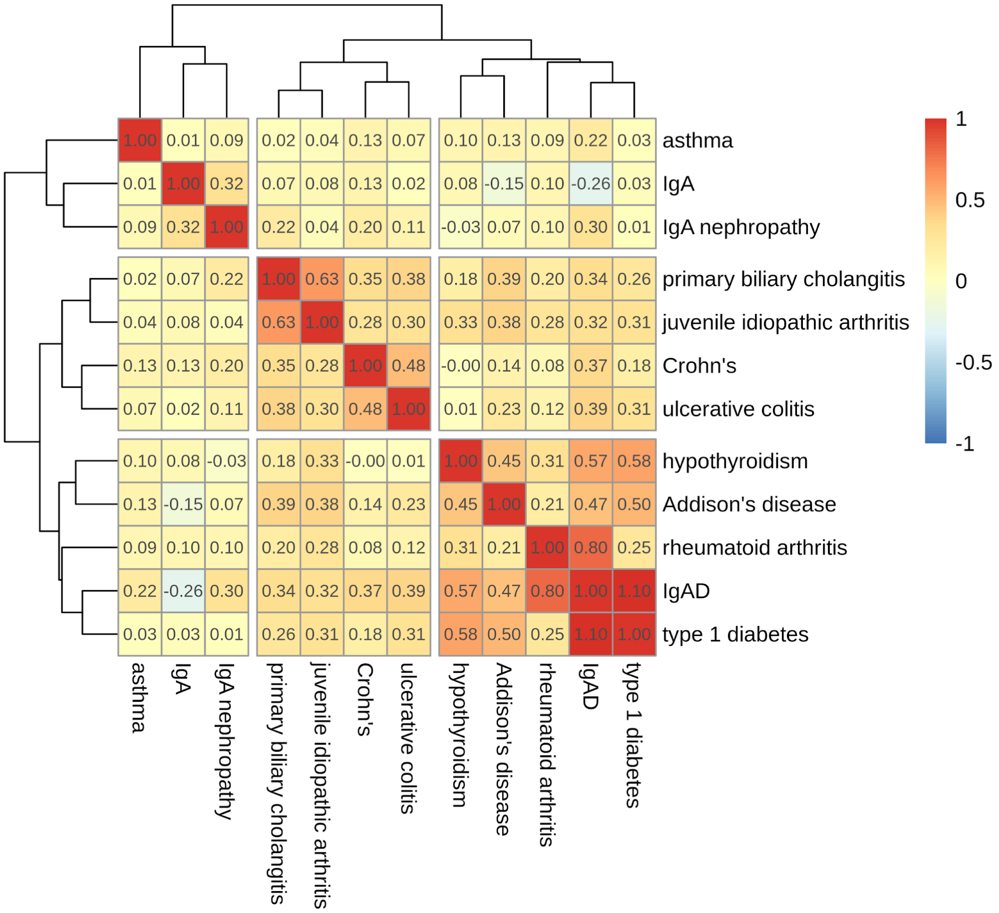

Supplementary Figure 9. Genetic correlation estimates among SIgAD ('IgAD') and selected immune traits. The traits were hierarchically clustered into three groups on the basis of our choice of three conditioning traits for the cFDR analysis.

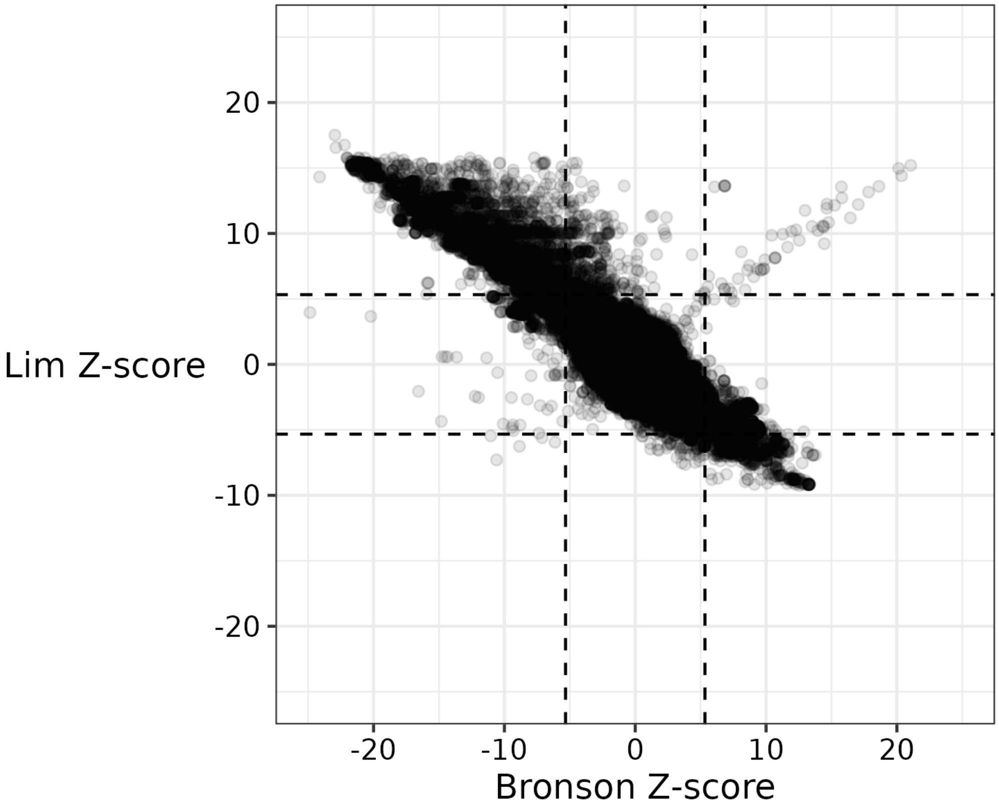

Supplementary Figure 10. Each point corresponds to a SNP common to both Bronson and colleagues' SIgAD GWAS and our GWAS of the SIgAD genotype data published by Lim and colleagues. The x- and y-coordinate of each point are given by the Z-score of the SNP in the Bronson and Lim GWAS, respectively.

Supplementary data

Supplementary Data 1. IDs, names, and genomic coordinates of the 448 IEI genes used in enrichment analysis. *id* is the Ensembl gene ID. *tss* gives the position of the transcription start site. *fwdStrand* indicates whether the gene is found on the forward or reverse strand.

Supplementary methods

Detection and correction of mislabelled columns in the Bronson data set

We observed an apparent mislabelling of the two allele columns in the version of Bronson and colleagues' SIgAD GWAS data set available on the GWAS Catalog. This mislabelling reversed the labels of the 'effect' and 'other' allele columns without also taking the reciprocal of the odds ratio, with the consequence that the odds ratio estimate reported at each SNP was the multiplicative inverse of the true odds ratio estimate expected for the given alleles. We determined these columns were mislabelled after cross-referencing several sources of information relating to four non-MHC lead SNPs reported: the summary statistics themselves, the GWAS Catalog's summary of lead SNPs, and Table 2 and Supplementary Table 5 of Bronson and colleagues' paper (Supplementary Table 7). The Catalog summary reported the same odds ratio estimates as those in the summary statistics, but reported as risk (i.e. 'effect') alleles those alleles labelled 'other' alleles in the summary statistics.

In Supplementary Table 5 of their paper, Bronson et al. provided allele frequencies and odds ratios for these lead SNPs in each national subset making up the study cohort. The alleles were labelled only as 'A' and 'B', and the direction of the odds ratio with respect to these was not specified. We recomputed the allelic odds ratios using the information given in Supplementary Table 5 for the Swedish cohort (the largest national cohort) and used the resulting ratios to disambiguate the direction of the stated odds ratios. These transpired to have as their effect allele whichever was the minor allele in cases, such that neither 'A' nor 'B' was consistently designated the effect allele for the stated odds ratios. We found that odds ratios with allele 'B' as the effect allele matched the direction of effect for the odds ratios reported in the Catalog summary and the summary statistics, and that allele 'B' matched the Catalog summary's reported risk allele but the summary statistics' 'other' allele. These findings (restricted as they are to only four variants) are consistent with the summary statistics' allele column labels having been reversed without a concomitant modification of the corresponding odds ratios.

We corroborated these findings by performing a GWAS of SIgAD using genotype data published by Lim and colleagues [182] on the European Nucleotide Archive under project accession PRJEB4929. The cases in this cohort appear to correspond to a subset of the cohort

studied by Bronson and colleagues, although this apparent overlap was not made explicit in Lim et al. We made this determination on the basis of the similarity in the reported provenance of the samples and the genotyping technologies used. Access to these genotype data meant we were able to unambiguously determine the ‘effect’ and ‘other’ allele in the GWAS regression. After processing the summary statistics from this GWAS as described above, we compared the effect direction at each SNP also present in Bronson and colleagues’ data set and found that the direction of effect was consistently reversed in the latter relative to the former (Supplementary Figure 10).
